# Supplementary material for: Development of a Rapid Gold Nanoparticle Immunochromatographic Strip Based on the Nanobody for Detecting 2,4-DichloRophenoxyacetic Acid
Source: Biosensors (Basel). 2022 Jan 30;12(2):84. doi: 10.3390/bios12020084 (PMC8869386; doi:10.3390/bios12020084)
Supplement: Supplementary file 1 [file biosensors-12-00084-s001.zip › biosensors-1550820-supplementary.pdf]

Supplementary Materials

Development of a Rapid Gold Nanoparticle Immunochromatographic Strip Based on the Nanobody for Detecting 2,4-Dichlo-Rophenoxyacetic Acid

Hui Zhou [1,†], Cong He [1,†], Zhenfeng Li [2], Jingqian Huo [1,*], Yu Xue [1], Xiaotong Xu [1], Meng Qi [1,2], Lai Chen [1], Bruce D. Hammock [2] and Jinlin Zhang [1,*]

1 College of Plant Protection, Hebei Agricultural University, Baoding 071001, China; zhouhui970618@163.com (H.Z.); hecong9696@163.com (C.H.); xy1135723614@163.com (Y.X.); xxt20011127@163.com (X.X.); menqi@ucdavis.edu (M.Q.); chenlai@hebau.edu.cn (L.C.)

2 Department of Entomology and Nematology and UCD Comprehensive Cancer Center, University of California, Davis, CA 95616, USA; lizhenfeng306@126.com (Z.L.); bdhammock@ucdavis.edu (B.D.H.)

* Correspondence: huojingqian@hebau.edu.cn (J.H.); zhangjinlin@hebau.edu.cn (J.Z.)

† These authors contributed equally to this work.

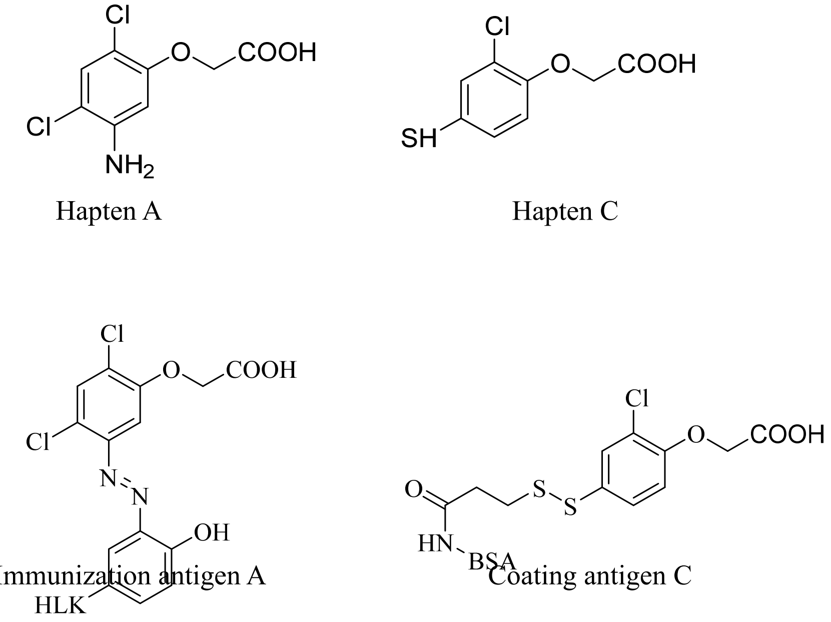

Figure S1. Structure of immunization antigen and coating antigen.

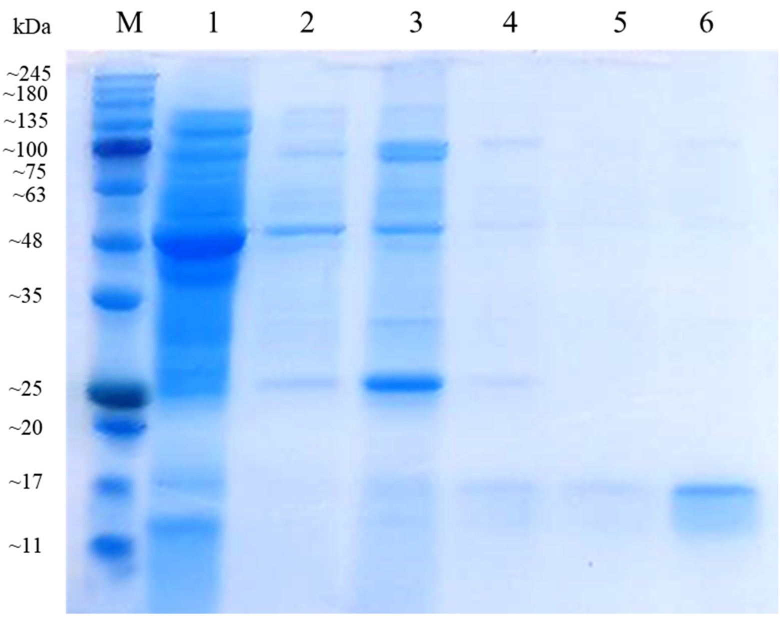

Figure S2. Sodium dodecyl sulfate polyacrylamide gel electrophoresis (SDS-PAGE) analysis of the expression of 2,4-D nanobody. Key: lane M, the rainbow 245 Broad Spectrum Protein Marker (11-245 KD); lane1, the wash buffer; lane2, 25 mM imidazole 1; lane3, 25 mM imidazole 2; lane4, 50 mM imidazole 1; lane5, 50 mM imidazole 2, lane6, 100 mM imidazole. When the concentration of imidazole reached 100 mM, the protein was eluted, and whose size was about 17 kDa.

(a)

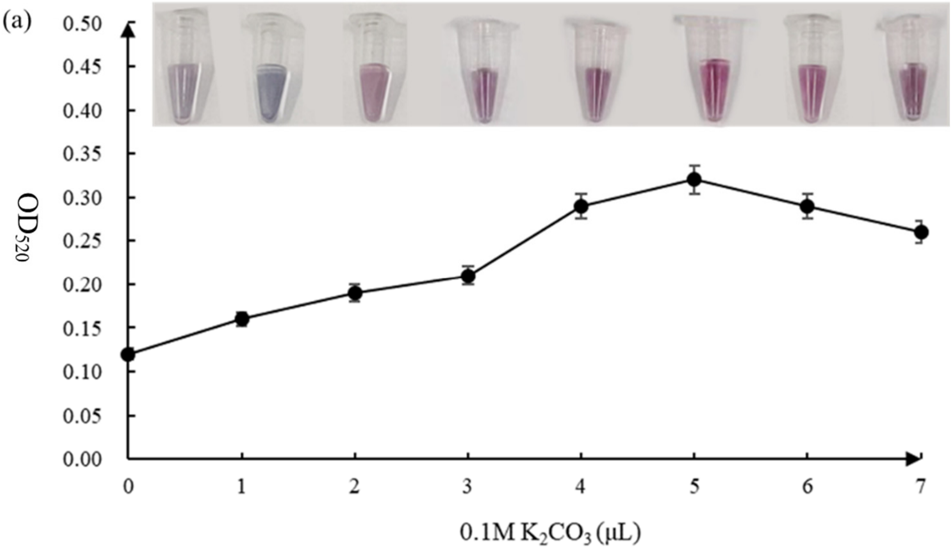

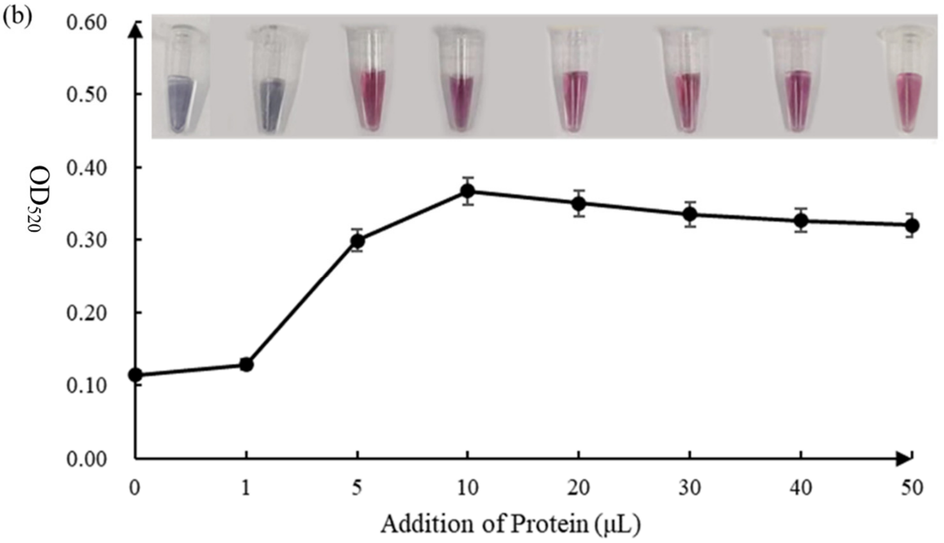

Figure S3. Absorbance curve for optimal pH value and nanobody amount. (**a**) Optimization of the pH for conjugation. (**b**) Optimization of the nanobody amount for conjugation.

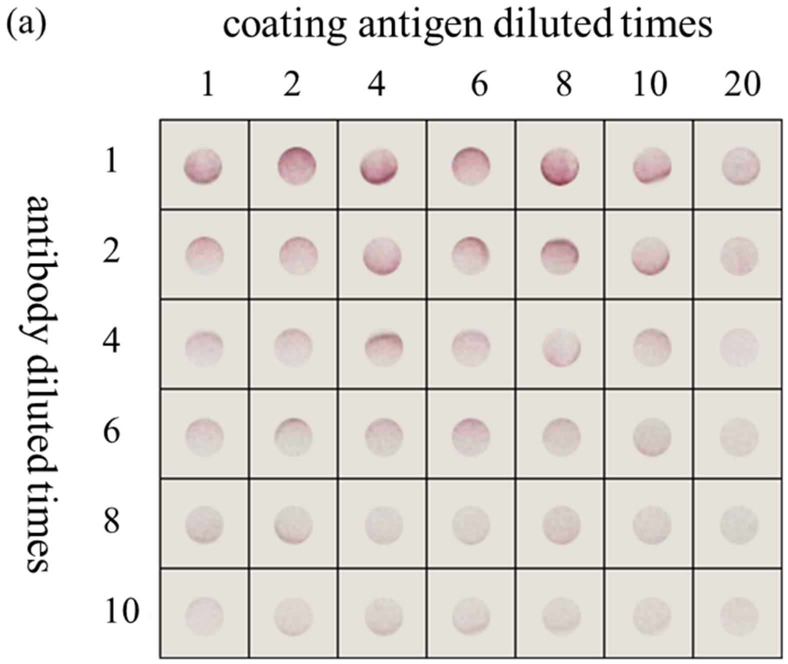

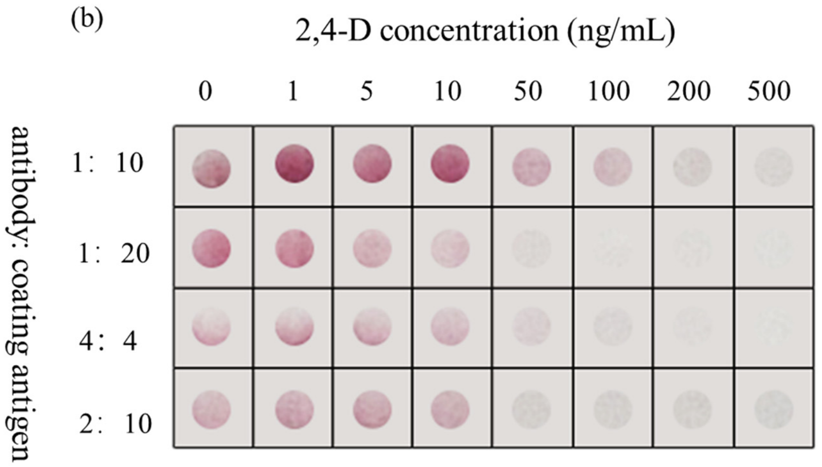

Figure S4. Screen the best combination of antibody and coating antigen. (**a**) Screen the better combinations of antibody and coating antigen using the checkerboard method. (**b**) Screen the best combination of antibody and coating antigen using the competition method.

Preparation of immunogen and coating antigens [1]

Diazotization method for preparation of immunogen

Followed the previous procedure, sodium nitrite (1%, 175 μL) was added slowly to a solution of Hapten A (5 mg) in 2 M HCl (2 mL) in an ice-cold bath, and the mixture was stirred for 30 min. The mixture was added dropwise into 6 mL of phosphate buffer saline (PBS) solution containing KLH (60 mg). The pH was kept between 6-8 by adding 2 M KOH. After that, the mixture reacts for 12 h at 4 °C to prepare the immunogen. The resulting conjugates were dialyzed in PBS at 4 °C and stored at -20 °C.

SPDP method for preparation of coating antigen

Followed the previous procedure, hapten C was coupled to carrier protein BSA by the heterobifunctional agent 3-(2-pyridyldithio) propionic acid N-hydroxysuccinimide ester (SPDP). SPDP (9 mg) in 300 μL of DMSO was added to a solution of Hapten C (6 mg) and BSA (30 mg) dissolved in 2 mL of PBS, and the solution was mixed for 12 h at 4 °C to prepare coating antigen. The conjugates were dialyzed in PBS at 4 °C and stored at −20 °C.

Reference

1. Li, Z.F.; Dong, J.X.; Vasylieva, N.; Cui, Y.L.; Wan, D.B.; Hua, X.D.; Huo, J.Q.; Yang, D.C.; Gee, S.J.; Hammock, B.D. Highly specific nanobody against herbicide 2,4-dichlorophenoxyacetic acid for monitoring of its contamination in environmental water. *Sci Total Environ*. **2020**, *753*, 141950. https://doi.org/10.1016/j.scitotenv.2020.141950.
